# Supplementary figures and images for: Trade-Offs between Competitive Ability and Resistance to Top-Down Control in Marine Microbes
Source: mSystems. 2023 Mar 14;8(2):e01017-22. doi: 10.1128/msystems.01017-22 (PMC10134844; doi:10.1128/msystems.01017-22)

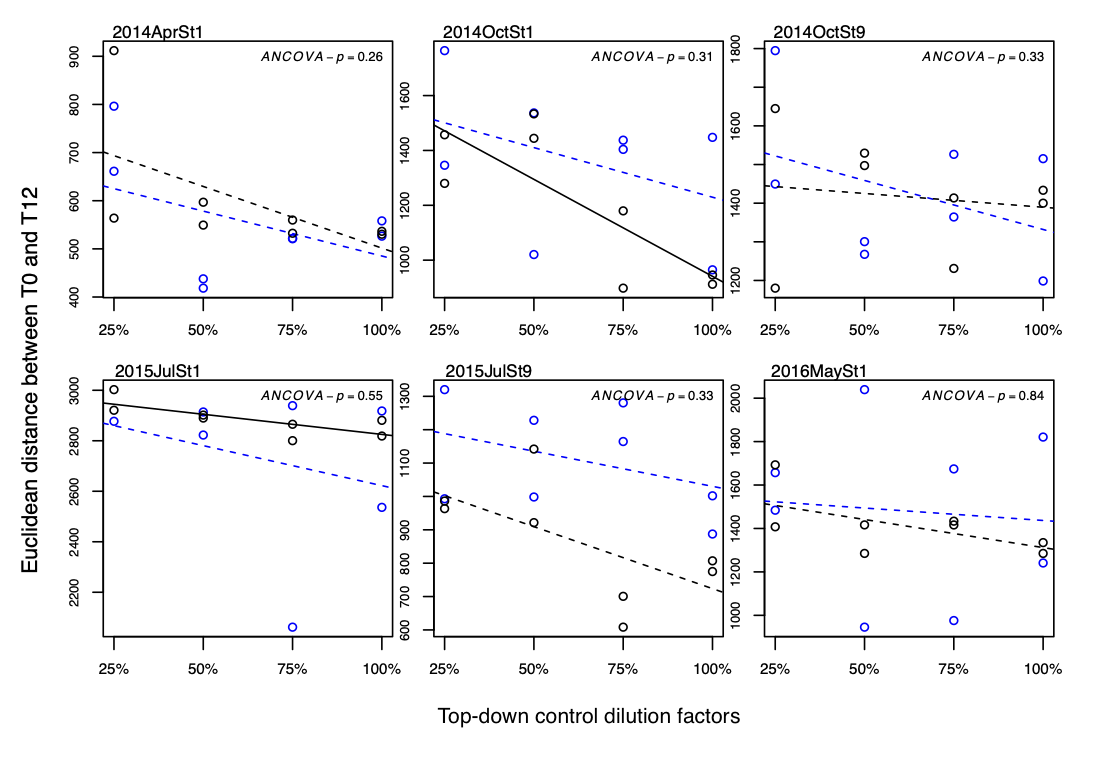

Supplement: FIG S3 [file msystems.01017-22-s0005.tif]

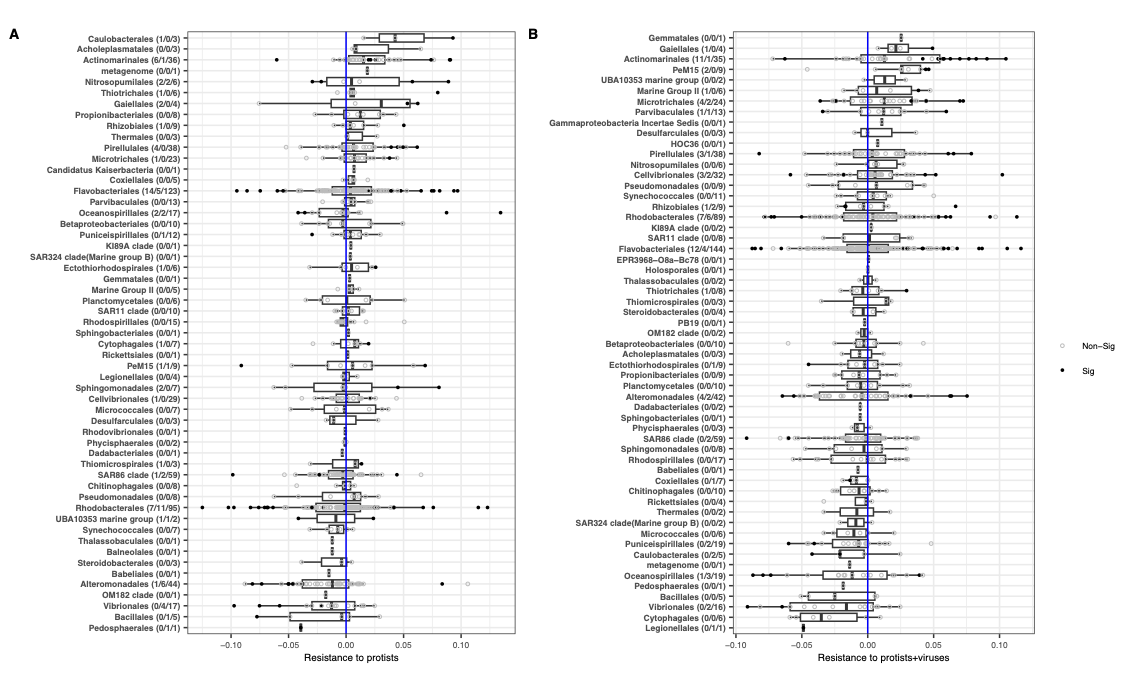

Supplement: FIG S4 [file msystems.01017-22-s0006.tif]

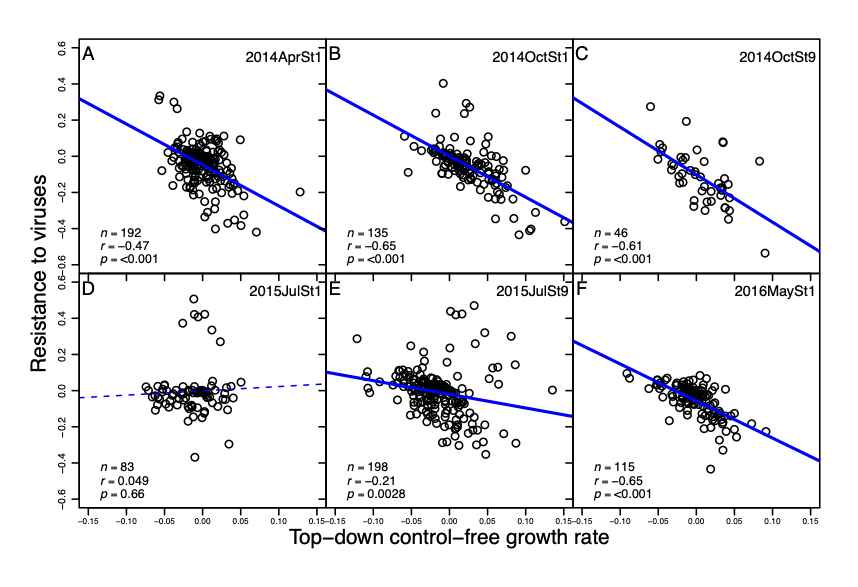

Supplement: FIG S5 [file msystems.01017-22-s0007.tif]

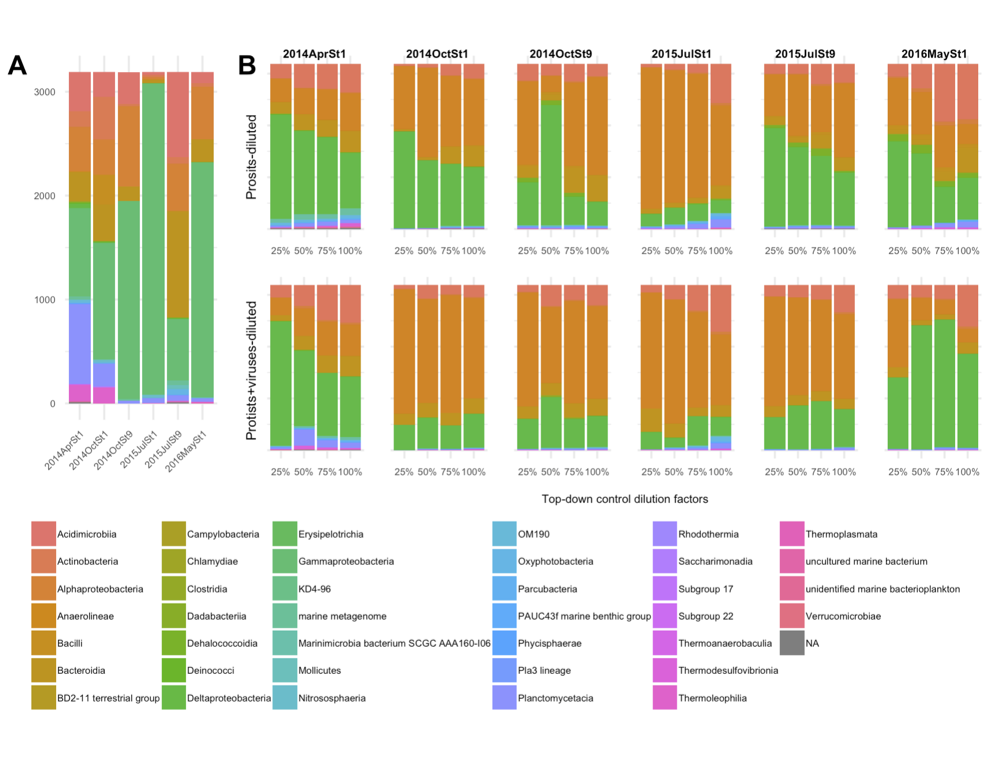

Supplement: FIG S6 [file msystems.01017-22-s0008.tif]

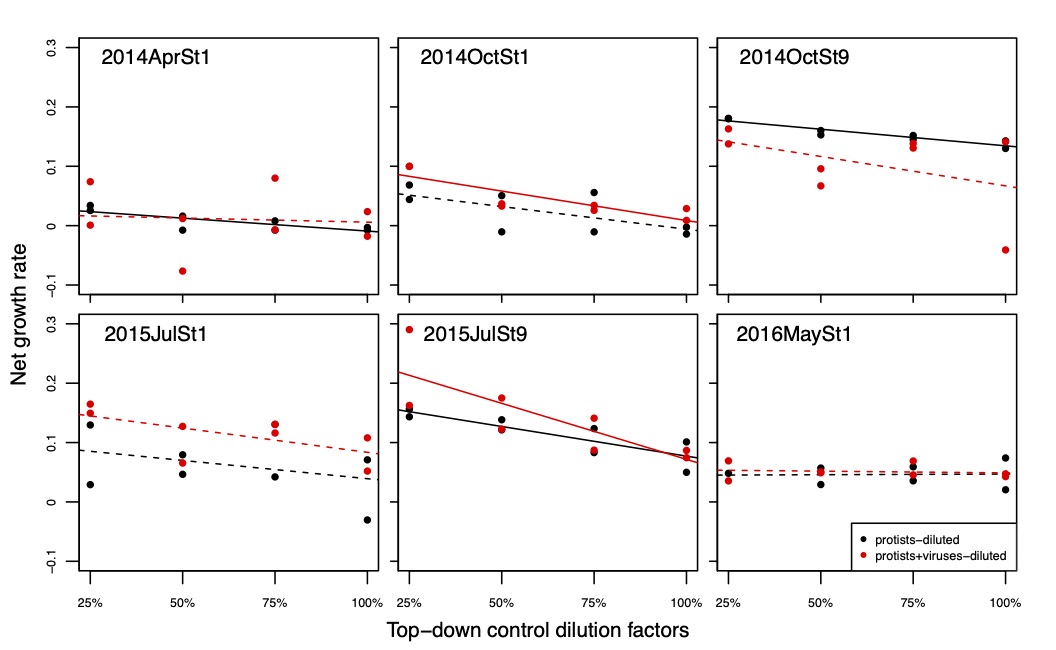

Supplement: FIG S7 [file msystems.01017-22-s0009.tif]

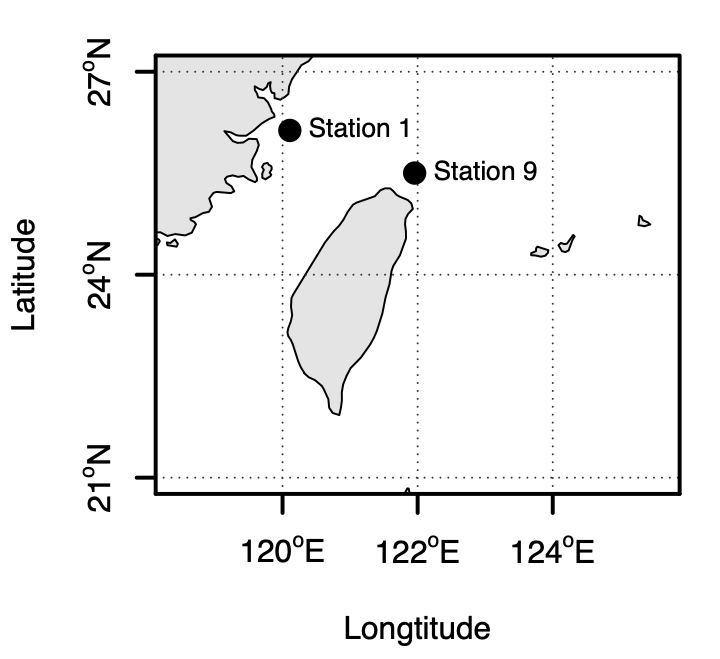

Supplement: FIG S1 [file msystems.01017-22-s0003.tif]

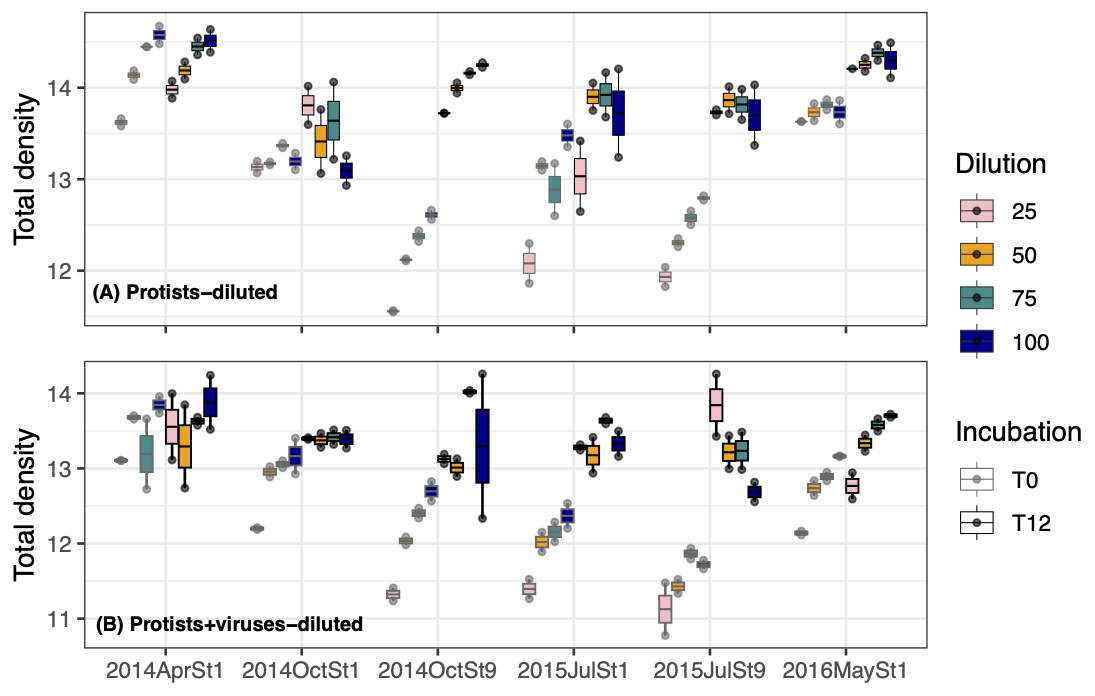

Supplement: FIG S2 [file msystems.01017-22-s0004.tif]
