# Supplementary material for: Trade-Offs between Competitive Ability and Resistance to Top-Down Control in Marine Microbes
Source: mSystems. 2023 Mar 14;8(2):e01017-22. doi: 10.1128/msystems.01017-22 (PMC10134844; doi:10.1128/msystems.01017-22)
Supplement: TABLE S1 [file msystems.01017-22-s0001.doc]

Table S1. Environmental and biotic variables and data information in each experiment. Bacterial initial richness was the number of ASV estimated from communities at T_0_ that rarefied with the lowest number of all samples in this study (at 3188 reads). Bacterial initial density was estimated by the means of bacterial density without dilution (100% top-down control dilution factor) at T_0_. The lowest richness values at T_12_ were estimated to provide appropriate scaling for RAD decay coefficient and evenness with controlled richness.

| **Experiment** | **Temperature  (°C)** | **NO_2_+NO_3_  (μM)** | **PO_4_  (μM)** | **Initial bacterial richness** | **Initial bacterial density (cells/ml)** | **Lowest number of sample reads** | **Lowest richness values at T_12_ (for scaling RAD and evenness)** |
| --- | --- | --- | --- | --- | --- | --- | --- |
| 2014AprSt1 | 16.86 | 16.41 | 0.46 | 327 | 1592878.56 | 6754 | 96 |
| 2014OctSt1 | 26.61 | 13.92 | 0.23 | 202 | 537658.85 | 7140 | 57 |
| 2014OctSt9 | 23.62 | 1.69 | 0.09 | 52 | 314478.76 | 3188 | 136 |
| 2015JulSt1 | 25.48 | 7.29 | 0.58 | 103 | 479109.83 | 12355 | 78 |
| 2015JulSt9 | 26.02 | 0.20 | 0.1 | 287 | 241740.23 | 6467 | 59 |
| 2016MaySt1 | 20.99 | 10.9 | 0.21 | 166 | 724227.43 | 13343 | 51 |
