# Supplementary material for: Trade-Offs between Competitive Ability and Resistance to Top-Down Control in Marine Microbes
Source: mSystems. 2023 Mar 14;8(2):e01017-22. doi: 10.1128/msystems.01017-22 (PMC10134844; doi:10.1128/msystems.01017-22)
Supplement: TABLE S2 [file msystems.01017-22-s0002.doc]

Table S2. Permutation test for analyzing the relationship between competitive ability (i.e., top-down control free growth rates) versus resistance (i.e., the inverse of mortality rates) to (A) protist grazing and (B) protists gazing and viral lysis combined, respectively. A null model was generated using the linear regression estimates with randomly shuffled dependent variables (resistance to top-down control) and repeated 1000 times. The p-value of the Shapiro-Wilk test was estimated to test if the null model was not significantly different from the normal distribution (where S-W p-value > 0.05). Z-score and its p-value were estimated to test if the observed value was significantly different from the mean of the null model.

| **Experiment** | **S-W p-value** | **z-score** | **p-value** |
| --- | --- | --- | --- |
| **(A) Protists-diluted treatment** | | | |
| 2014AprSt1 | 0.014 | -10.222 | < 0.001 |
| 2014OctSt1 | 0.470 | -4.189 | < 0.001 |
| 2014OctSt9 | 0.241 | -1.885 | 0.059 |
| 2015JulSt1 | 0.001 | -6.349 | < 0.001 |
| 2015JulSt9 | 0.363 | -9.091 | < 0.001 |
| 2016MaySt1 | 0.683 | -7.138 | < 0.001 |
| **(B) Protists+viruses-diluted treatment** | | | |
| 2014AprSt1 | 0.991 | -8.912 | < 0.001 |
| 2014OctSt1 | 0.649 | -6.431 | < 0.001 |
| 2014OctSt9 | 0.593 | -4.836 | < 0.001 |
| 2015JulSt1 | 0.568 | -5.899 | < 0.001 |
| 2015JulSt9 | 0.029 | -6.736 | < 0.001 |
| 2016MaySt1 | 0.394 | -7.638 | < 0.001 |
