# Supplementary material for: Trade-Offs between Competitive Ability and Resistance to Top-Down Control in Marine Microbes
Source: mSystems. 2023 Mar 14;8(2):e01017-22. doi: 10.1128/msystems.01017-22 (PMC10134844; doi:10.1128/msystems.01017-22)
Supplement: TEXT S1 [file msystems.01017-22-s0010.docx]

*Amplification mixture and PCR condition for 16S rRNA gene library preparation*

The V5–V6 region of the 16S rDNA was amplified using the forward primer FIA-787F (5’-[forward index adaptor]-ATTAGATACCCNGGTAG-3’) and reverse primer RIA-1046R (5’-[reverse index adaptor]-CGACAGCCATGCANCACCT-3’) (Cai *et al.,* 2013). The amplification mix contained: 1 U of *Taq* DNA polymerase (Promega), 1 × reaction buffer, 1.5 mM MgCl_2_, 0.2 mM dNTPs, 0.2 mM of primers, and 2 ng DNA. The PCR conditions were an initial denaturation at 94 °C for 3 min; 25 cycles of 94 °C for 30 s, 55 °C for 45 s, 72 °C for 1 min; and a final extension at 72 °C for 2 min. Three PCR amplifications were pooled and purified using AMPure XP beads (Beckman Coulter Genomic, CA, USA). Purified products were quantified using a Qubit fluorometer (Invitrogen, Carlsbad, CA, USA) with Qubit dsDNA BR Assay Kit (Life Technologies, USA).

A second PCR was performed using primers containing sample-specific indices and Illumina adaptors. The PCR mix contained: 1 U of *Taq* DNA polymerase (Promega), 1 × reaction buffer, 1.5 mM MgCl_2_, 0.2 mM dNTPs, 0.2 mM S5 and N7 primers (Nextera Index Kit) and 2 ng DNA purified from the first PCR product. The PCR conditions were an initial denaturation at 94 °C for 3 min; 6 cycles of 94 °C for 30 s, 55 °C for 45 s, 72 °C for 1 min; and a final extension at 72 °C for 2 min. Three PCR amplifications for each sample were pooled and purified using AMPure XP beads according to the manufacturer’s instructions. Final products with unique dual-index were quantified using a Qubit fluorometer and pooled in equal concentrations.

Reference: Cai L, Ye L, Tong AH, Lok S, Zhang T. 2013. Biased diversity metrics revealed by bacterial 16S pyrotags derived from different primer sets. PloS one. 14;8(1):e53649.
